# Efficacy and safety of sintilimab plus XELOX as a neoadjuvant regimen in patients with locally advanced gastric cancer: A single-arm, open-label, phase II trial

**DOI:** 10.3389/fonc.2022.927781

**Published:** 2022-08-26

**Authors:** Honghai Guo, Ping’an Ding, Chenyu Sun, Peigang Yang, Yuan Tian, Yang Liu, Scott Lowe, Rachel Bentley, Yaru Li, Zhidong Zhang, Dong Wang, Yong Li, Qun Zhao

**Affiliations:** ^1^ The Third Department of Surgery, The Fourth Hospital of Hebei Medical University, Shijiazhuang, China; ^2^ Internal Medicine, AMITA Health Saint Joseph Hospital Chicago, Chicago, Illinois, United States; ^3^ College of Osteopathic Medicine, Kansas City University, Kansas City, MO, United States; ^4^ Internal Medicine, Swedish Hospital, Chicago, IL, United States; ^5^ College of Osteopathic Medicine, Des Moines University, Des Moines, IA, United States

**Keywords:** neoadjuvant regimen, sintilimab, immunotherapy, locally advanced gastric cancer, phase II trial

## Abstract

**Background:**

Neoadjuvant chemotherapies have been widely recommended in patients with locally advanced gastric cancer (LAGC). However, the evidence of combining neoadjuvant chemotherapy with anti–programmed death 1 (anti–PD-1) antibody therapy for patients with LAGC is lacking. Thus, we conducted a single-arm phase II trial to evaluate the efficacy and safety of the anti–PD-1 antibody sintilimab plus XELOX regimen (capecitabine plus oxaliplatin) in patients with LAGC.

**Methods:**

Patients with LAGC (cT3-4 N+ M0, CY0, P0) were enrolled and received four preoperative cycles of sintilimab (200 mg, IV, Q21d) plus XELOX (oxaliplatin 130 mg/m^2^, IV, d1 with capecitabine 1,000 mg/m^2^, bid, d1–d14, Q21d) therapy. The primary endpoint was the pathological complete response (pCR) rate. This clinical trial was registered at Chictr.org.cn (trial number: ChiCTR2000030414).

**Results:**

Thirty patients were enrolled from March 2020 to July 2021, with a median age of 62 years (range, 30–72), and 18 (60.0%) were men. There were 19 (63.3%) patients with PD-L1 CPS ≥1.The pCR rate was 33.3% [95% confidence interval (CI), 17.3%–52.8%], and the major pathologic response (MPR) rate was 63.3% (95% CI, 43.9%–80.1%). All the patients underwent R0 resection. The objective response rate (ORR) and the disease control rate (DCR) were 70.0% (95% CI, 50.6%–85.3%) and 100% (95% CI, 88.4%–100%), respectively. Downstaging of the overall TNM stage was observed in 22 (73.3%) patients. The pCR rate in patients with PD-L1 CPS ≥1 and patients with PD-L1 CPS <1 was 42.1% vs. 18.2% (*P =* 0.246), whereas the MPR rate was 78.9% vs. 36.4% (*P =* 0.047). The potential immune-related adverse events (irAEs) were hypothyroidism (3.3%), pneumonia (10.0%), and dermatitis (6.7%). Grade3 common treatment-related adverse events (TRAEs) were ALT increase (3.3%), AST increase (3.3%), and dermatitis (3.3%) during the neoadjuvant therapy. There were no severe complications or death related to the surgery.

**Conclusion:**

Sintilimab plus XELOX as neoadjuvant therapy showed an encouraging pCR rate, MPR rate, and manageable safety. This combination of regimens might provide a new option for patients with LAGC.

**Clinical Trial Registration**: Chictr.org.cn, identifier ChiCTR2000030414.

## Introduction

Gastric cancer is the fifth most common cancer and the third leading cause of cancer-related deaths worldwide (1). In China, most patients with gastric cancer are in the advanced stage at the time of diagnosis with a dismal prognosis despite radical surgery (2–4). Recently, many randomized clinical trials (5–7) showed that neoadjuvant chemotherapy could increase R0 resection and tumor downstaging rates and improve long-term prognosis of patients with locally advanced gastric cancer (LAGC). Thus, neoadjuvant chemotherapy is recommended as a standard treatment of LAGC (8, 9). In Asia, doublet chemotherapy of cisplatin or oxaliplatin combined with 5-fluorouracil, capecitabine, or S-1 is a prior choice in clinical practice (7, 10, 11). Our previous studies found that neoadjuvant chemotherapy containing XELOX regimen (oxaliplatin and capecitabine) for LAGC effectively improved the R0 resection rate and prognosis (12, 13). However, because of the heterogeneity of gastric cancer, the pathological complete response (pCR) rate of chemotherapy is only between 4% and 16% (12–16). New therapeutic agents in addition to chemotherapy to improve the efficacy still need to be investigated.

Immune checkpoint inhibitors (ICIs) targeting programmed death-1 (PD-1) and PD-ligand 1 (PD-L1) showed promising efficacy in multiple malignancies, including gastric cancer. Combining ICIs and standard chemotherapy might exert synergistic antitumor activity by regulating the immune system and reshaping the tumor microenvironment, which improves survival in several cancer types (17–19). The KEYNOTE-649 study (20) and ATTRACTION-4 trial (21) revealed that anti–PD-1 antibody combined with chemotherapy improved overall survival (OS) and progression-free survival of patients with advanced or recurrent gastric cancer with acceptable safety. Sintilimab (Innovent Biologics, Suzhou, China) is a highly selective, humanized, and monoclonal antibody that blocks the interaction between PD-1 and its ligands. A phase III study demonstrated that the combination of sintilimab and chemotherapy had significant OS benefits and well-manageable toxicity in advanced gastric cancer (22).

However, the evidence of combining neoadjuvant chemotherapy with anti–PD-1 antibody in patients with LAGC is lacking. Thus, we designed a single-arm phase II trial to evaluate the efficacy and safety of the sintilimab plus XELOX regimen in patients with LAGC.

## Materials and methods

### Study design and participants

This study was a prospective clinical trial of sintilimab immunotherapy combined with XELOX chemotherapy in LAGC and was registered at Chictr.org.cn (trial number: ChiCTR2000030414). All eligible patients signed the informed consent according to the institutional and federal guidelines. The study design was approved by the Ethics Committee of the Fourth Hospital of Hebei Medical University with approval number 2019125.

The inclusion criteria were as follows: I) patients with the age of 18–75 years; II) patients having HER-2–negative gastric adenocarcinoma confirmed by histopathology; III) patients with a clinical stage of cT3/4aN+M0 evaluated by CT and laparoscopy; IV) patients with no prior antitumor therapy, including chemotherapy, radiotherapy, targeted therapy, or immunotherapy; V) patients with the Eastern Cooperative Oncology Group (ECOG) performance status score of ≤2; and VI) patients with adequate bone marrow, liver, heart, and kidney functions. The exclusion criteria were as follows: I) patients with positive peritoneal cytology (CY1); II) patients with laparoscopy confirmed peritoneal metastasis (P1); III) patients who had difficulty in self-administering oral medication due to gastrointestinal obstruction; IV) patients with other serious immunosuppressive conditions or simultaneous malignant tumors; V) patients with complications of severe uncontrolled infection or other serious uncontrolled concomitant disease; and VI) patients who had other comorbidities endangering the patient safety or affecting the study completion that was determined by the investigator.

### Treatment

Eligible patients received neoadjuvant therapy with XELOX regimen (oxaliplatin 130 mg/m^2^ *via* intravenous infusion on day 1 with capecitabine 1,000 mg/m^2^ oral twice daily on days 1 to 14 of each cycle) and sintilimab intravenous infusion at a dose of 200 mg over 1 h, for four cycles (21 days per cycle).

The patients were evaluated using abdominal-pelvic CT, 4 weeks after the completion of four cycles of neoadjuvant therapy. If the tumor progressed during the neoadjuvant period, then the treatment was discontinued and the surgery or other appropriate anticancer treatment was administrated according to the investigator’s discretion. Before surgery, a second peritoneal check by laparoscopy was done to further exclude occult peritoneal metastasis. The objective of surgery was for R0 resection, defined as curative resection of gastric primary lesions and regional lymph nodes without residual tumor cells at the margin of resection. An open gastrectomy, laparoscopic gastrectomy, or robotic gastrectomy was selected according to the nature of the patient and the expertise of the surgeon. Surgical procedures consisted of either 1) proximal subtotal gastrectomy (with jejunal interposition surgery or an esophageal gastric remnant anastomosis), 2) distal subtotal gastrectomy (with gastrojejunostomy and Braunth II anastomosis), or 3) total gastrectomy (with jejunal interposition surgery) and D2 lymphadenectomy. Patients received another four cycles of XELOX regimen 1 month after the operation.

### Assessments

The primary endpoint was the pCR rate. The secondary endpoints were the major pathologic response (MPR) rate, disease control rate (DCR), objective response rate (ORR), R0 resection rate, tumor downstaging, safety, disease-free survival (DFS), and OS. The pathological response of the tumor to neoadjuvant sintilimab plus XELOX was evaluated. The resection margin status and tumor regression grade (TRG) were reported. TRG was defined as follows (8): Grade 0, complete remission (no cancer cells); Grade 1, partial remission (single cells or small groups of cancer cells); Grade 2, low efficiency (residual cancer outgrown by fibrosis); and Grade 3, poor efficiency (minimal or no treatment effect and extensive residual cancer cells). Achievement of TGR 0 was defined as having pCR. MPR was defined as having <10% residual tumor cells.

Tumor response was evaluated on the basis of the Response Evaluation Criteria In Solid Tumors [RECIST version 1.1 (23)] and categorized as follows: complete response (CR), partial response (PR), stable disease (SD), and progressive disease (PD). The sum of CR and PR was defined as ORR. The sum of CR, PR, and SD was defined as DCR. Tumor staging (cTNM and ypTNM) was performed on the basis of the criteria developed by American Joint Committee on Cancer (eight edition). The successful downstaging was defined as the decrease of clinical TNM stage of patients from stage III to stage II or I after neoadjuvant sintilimab plus XELOX therapy.

Tumor tissue samples for analyzing PD-L1 expression were obtained surgically. Collected tumor specimens were formalin-fixed and paraffin-embedded and then cut into 4- to 5-µm-thick sections for further staining. The primary antibody used was anti–PD-L1 (IHC 22C3 pharmDx, Dako North America Inc., Carpinteria, CA, USA). A combined positive score (CPS) was defined as the number of all positive staining cells including tumor cells, lymphocytes, and macrophages, divided by the total number of viable tumor cells and multiplied by 100.

Surgical safety was assessed by the incidence of surgery-related complications. During the treatment, all patients were monitored for toxicity, and the classification was according to the NCI Common Terminology Criteria for Adverse Events (CTCAE) version 5.0.

### Statistics

Considering the history of pCR rate of chemotherapy as between 4% and 16% (12–16), we chose 11% pCR for the null hypothesis. A sample size of 28 achieved 80.413% power to detect a target pCR rate of 30% using a two-sided exact test with a significance level of 0.05. Because the pCR was a short term indicator, a relatively low dropout rate was needed. Finally, a sample size of 30 patients was determined, allowing a 5% dropout rate.

SPSS version 26.0 and GraphPad Prism 9.0 were utilized to perform statistical analyses. The classified data were described by number and percentage, whereas the quantitative data were described by median with range. The pCR, MPR, DCR, ORR, and R0 resection rate with 95% CI were calculated using the Clopper–Pearson exact method based on binomial distribution. Fisher’s exact test was used for classification variables. Median follow-up time was calculated using the reverse Kaplan–Meier method. Survival was analyzed with the Kaplan–Meier method. *P*-value of <0.05 was considered statistically significant.

## Results

### Patient characteristics

**Figure 1:**
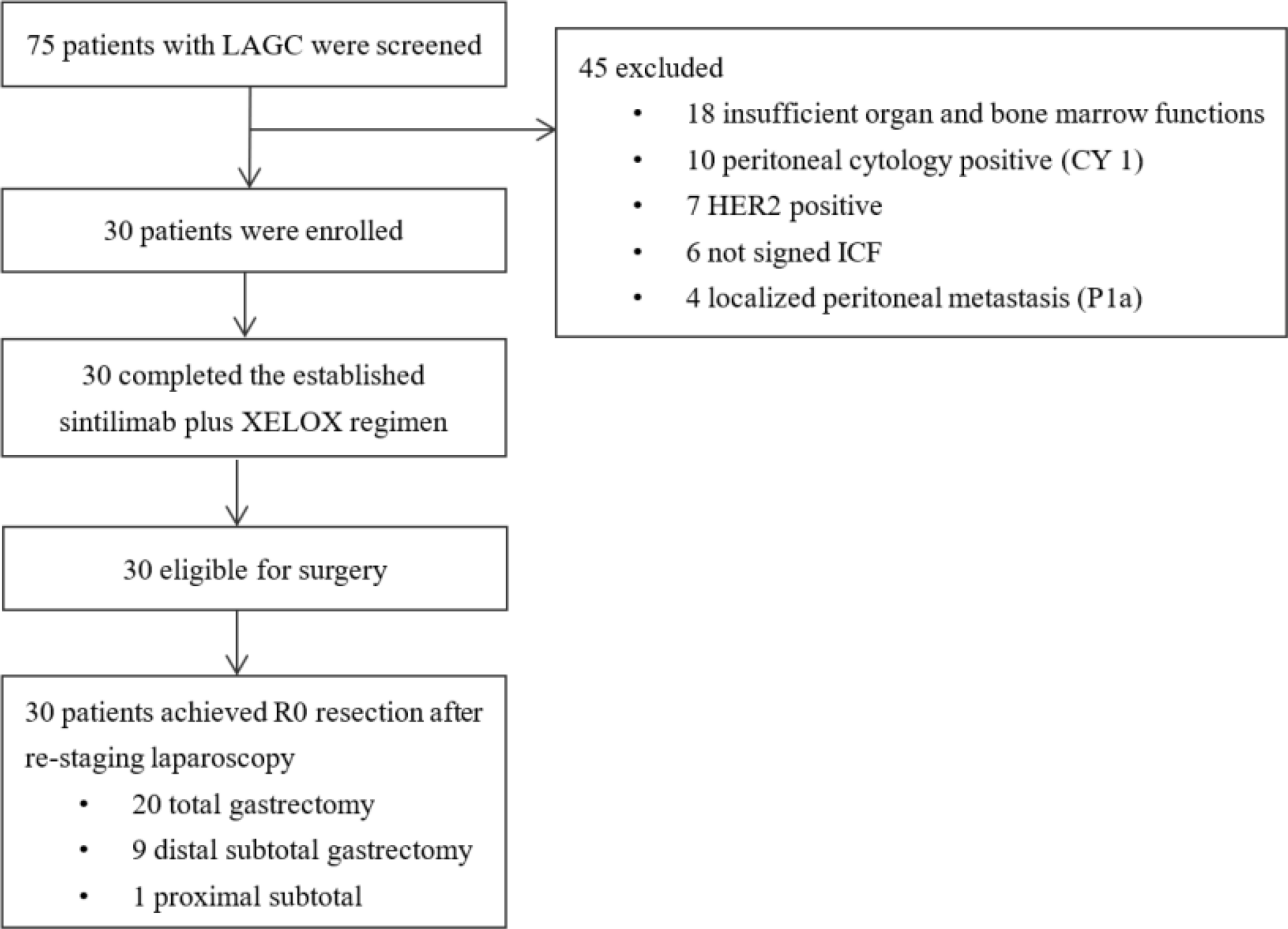
Study flow chart.

Fifty-one patients with LAGC were screened from March 2020 to July 2021. Among them, 21 patients were excluded including 10 patients for CY1, seven patients for HER-2 positive (3+ and 2+, fish+), and four patients for localized peritoneal metastasis (P1a) (**Figure 1**). Thirty eligible patients were subsequently enrolled. Baseline characteristics are listed in **Table 1**. The median age was 62 (range, 30–72 years), and 18 (60.0%) were men. The PD-L1 expression status was detected for all patients and 19 cases (63.3%) were PD-L1 positive (CPS ≥ 1).

**Table 1:** Baseline demographic and patient characteristics.

Characteristics	patients (N = 30)
Age (years)	
Median (Range)	62 (30–72)
Gender, No. (%)	
Male	18 (60.0)
Female	12 (40.0)
ECOG score, No. (%)	
0	19 (63.3)
1	8 (26.7)
2	3 (10.0)
Tumor size (cm)	
<5.0	16 (53.3)
≥5.0	14 (46.7)
Histological type, No. (%)	
Poorly differentiated	13 (43.3)
Moderately differentiated	5 (16.7)
Well differentiated	12 (40.0)
Clinical T stage, No. (%)	
cT3	8 (26.7)
cT4	22 (73.3)
Clinical N stage, No. (%)	
cN1	8 (26.7)
cN2	14 (46.7)
cN3	8 (26.7)
PD-L1 overexpression No. (%)	
CPS < 1	11 (36.7)
CPS ≧ 1	19 (63.3)
Clinical stage III, No. (%)	30 (100.0)

CPS, combined positive score; ECOG, Eastern Cooperative Oncology Group; PD-L1, programmed cell death ligand-1.

### Tumor response to neoadjuvant sintilimab plus XELOX therapy

**Figure 2:**
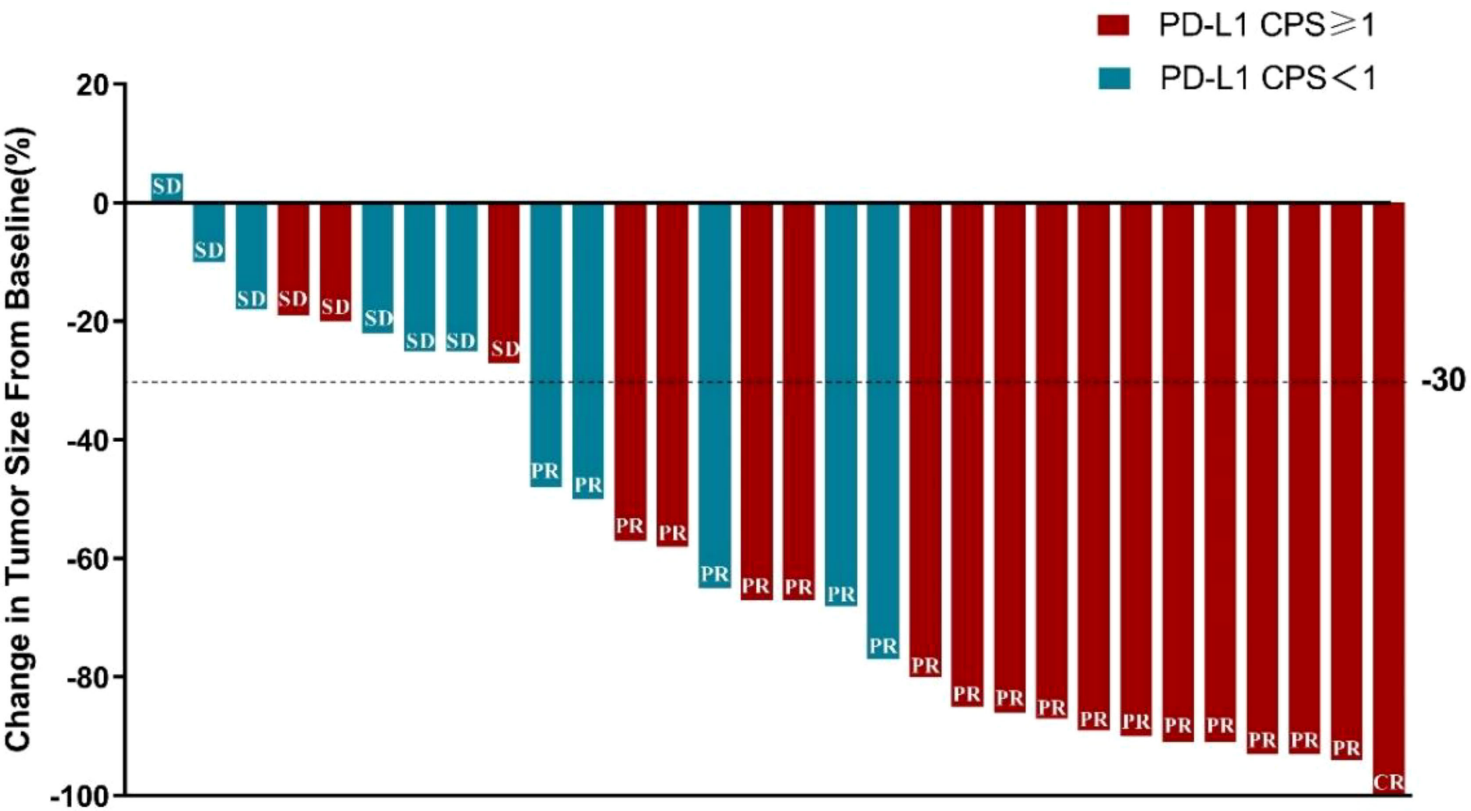
A waterfall plot of best ranked tumor reduction.

All 30 patients completed the neoadjuvant sintilimab plus XELOX treatment, and then, tumor response was assessed by CT scan. According to RECIST1.1 criteria, one patient (6.7%) achieved CR, 19 patients (63.3%) achieved PR, nine patients (30.0%) achieved SD, and no one had PD. The best ranked tumor reduction was presented by a waterfall plot (**Figure 2** **)**. The ORR and DCR were 70.0% (95% CI, 50.6%–85.3%) and 100% (95% CI, 88.4%–100.0%), respectively.

### Surgical treatment

All 30 patients underwent re-staging laparoscopy and underwent radical resection with D2 lymphadenectomy, including 20 (66.7%) patients of total gastrectomy, nine patients (30.0%) of distal gastrectomy, and one patient (3.3%) of proximal gastrectomy. The R0 resection rate was 100.0% (95% CI, 88.4%–100.0%). The median intraoperative blood loss was 50.0 ml (range, 10.0–200.0 ml). The median operative time was 215.0 min (range, 160.0–300.0 min). The median postoperative hospitalization time was 8.5 days (range, 6.0–23.0 days).

### Pathologic response and downstaging

All 30 patients underwent postoperative specimen evaluation. Among them, 10 (33.3%), nine (30.0%), six (20.0%), and five (16.7%) patients achieved TRG 0, 1, 2, and 3, respectively. The pCR rate was 33.3% (95% CI, 17.3%–52.8%). The proportion of patients with MPR was 63.3% (95% CI, 43.9%–80.1%). The pCR rate in patients with PD-L1 CPS ≥ 1 and patients with PD-L1 CPS < 1 was 42.1% vs. 18.2% (*P =* 0.018), whereas the MPR rate was 78.9% vs. 36.4% (*P =* 0.047). The pCR rate in patients with PD-L1 CPS ≥ 5 and patients with PD-L1 CPS < 5 was 66.7% vs. 19.0% (*P =* 0.246), whereas the MPR rate was 100.0% vs. 47.6% (*P =* 0.011) **(** **Table 2** **)**. Among the 30 patients, 20 (76.7%) patients had at least one grade decrease in T stage, and 19(63.3%) had reached N0. Twenty-two (73.3%) patients achieved a decrease of the overall TNM stage (**Table 3**). Among 14 patients who had MS status test, only one patient was deficient MMR (dMMR), with a high PD-L1 expression (CPS = 30), and achieved MPR.

**Table 2:** Clinicopathological results of 30 patients after surgery. No. (%).

Variable	CPS ≥ 1(N = 19)	CPS < 1(N = 11)	*P*-Value	CPS ≥ 5(N = 9)	CPS < 5(N = 21)	*P*-Value
R0 resection	19 (100)	11 (100)	–	9 (100)	21 (100)	–
TRG						
0	8 (42.1)	2 (18.2)	0.149^#^	6 (66.7)	4 (19.0)	0.028^#^
1	7 (36.8)	2 (18.2)		3 (33.3)	6 (28.6)	
2	2 (10.5)	4 (36.4)		0 (0)	6 (28.6)	
3	2 (10.5)	3 (27.3)		0 (0)	5 (23.8)	
pCR	8 (42.1)	2 (18.2)	0.246^#^	6 (66.7)	4 (19.0)	0.018^#^
MPR, No. (%)	15 (78.9)	4 (36.4)	0.047^#^	9 (100.0)	10 (47.6)	0.011^#^
ypTNM stage, No. (%)						
I	6 (31.6)	1 (9.1)	0.061^#^	3 (33.3)	4 (19.0)	0.010^#^
II	3 (15.8)	2 (18.2)		0 (0)	5 (23.8)	
III	2 (10.5)	6 (54.5)		0 (0)	8 (38.1)	
Inevaluable*	8 (42.1)	2 (18.2)		6 (66.7)	4 (19.0)	

TRG, tumor regression grade; pCR, pathological complete response; MPR, major pathological response; TNM, tumor node metastasis. *No viable tumor cells remain in the sections where tumor cells are likely to remain. ^#^Fisher exact test.

**Table 3:** The downgrade changes of TNM staging in 30 patients before and after neoadjuvant chemotherapy. No. (%).

Variable		Patients (N = 30)	
		Pre-neoadjuvant therapy(laparoscopy)	Post-neoadjuvant therapy(surgical pathology)
T stage			
0		0	10 (33.3)
1		0	4 (13.3)
2		0	4 (13.3)
3		8 (26.7)	5 (16.7)
4		22 (73.3)	7 (23.3)
N stage			
N0		0	19 (63.3)
N+		30 (100.0)	11 (36.7)
M stage			
M0		30 (100.0)	30 (100.0)
Change in overall stage			
Downstaged			22 (73.3)
Upstaged			0
No change			8 (26.7)

### Adjuvant treatment and survival outcomes

Twenty-seven patients completed the four cycles of adjuvant treatment. Three patients received three cycles of adjuvant treatment due to compliance. As of data cutoff, the median follow-up was 15.4 months (range, 11.4 to 26.3 months). The median DFS and OS were not reached, and the 1-year DFS rate was 92.9% (95% CI, 84.0%–100%). Four patients developed recurrence after surgery, with one hepatic and retroperitoneal lymph nodes metastasis, one hepatic metastasis, one lung metastasis, and one peritoneal cytology positive. All of them were ypTNM stage II or III. The patient with hepatic and retroperitoneal lymph nodes metastasis died 15.0 months after surgery.

### Safety

Among the 30 patients, 27 (90.0%) experienced treatment-related adverse events (TRAEs) of any grade during the neoadjuvant therapy period. The common TRAEs were anemia (36.7%), neutropenia (30.0%), leukopenia (36.7%), ALT increase (23.3%), AST increase (13.3%), thrombocytopenia (10.0%), vomiting (10.0%), and diarrhea (26.6%). The potential immune-related adverse events (irAEs) were hypothyroidism (3.3%), pneumonia (10.0%), and dermatitis (6.7%). Most of the TRAEs were of grade 1 or 2. Grade 3 TRAEs included ALT increase (3.3%), AST increase (3.3%), and dermatitis (3.3%) (**Table 4**). No treatment-related death occurred.

**Table 4:** Treatment-related adverse events [N = 30, No. (%)].

Toxic effects	Grade 1	Grade 2	Grade 3	Grade 4/5
Hematologic				
Leukopenia	7 (23.3%)	4 (13.3%)	0	0
Neutropenia	5 (16.7%)	4 (13.3%)	0	0
Anemia	9 (30.0%)	6 (6.7%)	0	0
Thrombocytopenia	3 (10.0%)	0	0	0
ALT increase	5 (16.7%)	1 (3.3%)	1 (3.3%)	0
AST increase	3 (10.0%)	0	1 (3.3%)	
Non-hematologic				
Vomiting	3 (10.0%)	0	0	0
Diarrhea	6 (20.0%)	2 (6.7%)	0	0
Hypothyroidism	0	1 (3.3%)	0	0
Pneumonia	3 (10.0%)	0	0	0
Dermatitis	1 (3.3%)	0	1 (3.3%)	0

ALT, alanine aminotransferase; AST, aspartate aminotransferase.

Surgery-related complications were observed in 11 patients (36.7%) including pneumonia (26.7%), pleural effusion (13.3%), chyle leakage (6.7%), anastomotic leakage (3.3%), and gastroparesis (3.3%) (**Table 5**). All of them were cured by conservative treatment. There were no patients who underwent reoperation and received intensive care unit stay or re-admission.

**Table 5:** Postoperative complications. No. (%).

Postoperative Complications	Patients (N = 30)
Pneumonia	8 (26.7)
Pleural effusion	4 (13.3)
Chyle leakage	2 (6.7)
Anastomotic leakage	1 (3.3)
Gastroparesis	1 (3.3)

## Discussion

Our study showed the short-term benefit of adding sintilimab to neoadjuvant chemotherapy in patients with LAGC. The study achieved the primary endpoint, pCR rate of 33.3%, which was higher than the expected rate. Notably, neoadjuvant immune-chemotherapy improved the MPR rate, which was greater in patients with PD-L1 positive than patients with PD-L1 negative.

At present, assessing pathological tumor response after neoadjuvant therapy might provide objective and precious information about treatment efficacy and patients’ outcome (24). The pCR was shown as an effective indicator of good short-term efficacy, and a good predictor of the recurrence, metastasis, and survival in patients with gastric cancer after neoadjuvant therapy (25–27). The pCR rate was 33.3%, which was higher than that (4.0%–6.3%) observed in several clinical studies (12–15) with neoadjuvant XELOX. A 16.0% pCR rate was observed in the FLOT-4 trial (16), the rates of 16.1% and 16.9% were observed in our previous studies with DOX (13) and preoperative chemoradiation plus neoadjuvant XELOX, respectively (28). Recently, another phase 2 study evaluating the efficacy and safety of sintilimab combined with CapeOx in the neoadjuvant setting also reported a pCR rate of 19.4%. In contrast to the three-cycle neoadjuvant regimen explored in this study, we explored the efficacy and safety of four-cycle neoadjuvant therapy with preoperative sintilimab, oxaliplatin, and capecitabine, which is also commonly used in clinical practice. However, both the results suggest that a trend of immune-chemotherapy may improve pCR (29). Studies demonstrated that chemotherapy drugs and radiation therapy modulated the immune status of the tumor microenvironment and promoted the release of tumor antigens (30–32). This approach may have resulted in a synergistic effect with ICIs in gastric cancer. This result may have been the reason for the achievement of an astonishing pCR rate and MPR rate by this new perioperative treatment regimen in this study.

Some studies with neoadjuvant therapy also demonstrated that PD-L1 expression was correlated with pCR (33–35). In our study, 19 patients had PD-L1 CPS ≥1. The pCR rates in patients with PD-L1 CPS ≥1 and PD-L1 CPS <1 were 42.1% and 18.2%, respectively. The MPR rates in patients with PD-L1 CPS ≥1 and PD-L1 CPS <1 were 78.9% and 36.4%, respectively. Nine patients had PD-L1 CPS ≥5. The pCR rates in patients with PD-L1 CPS ≥5 and PD-L1 CPS <5 were 66.7% and 19.0%, respectively. The MPR rates in patients with PD-L1 CPS ≥5 and PD-L1 CPS <5 were 100.0% and 47.6%, respectively. We found that the PD-L1 expression might enhance response to neoadjuvant sintilimab plus XELOX therapy. However, these results need more prospective clinical trials to confirm.

The incidence of peritoneal metastasis in LAGC is more than 20% (36–38). Hence, in addition to the standard diagnostic workup, all patients enrolled in this study underwent diagnostic laparoscopy and peritoneal lavage to confirm the absence of positive peritoneal cytology and peritoneal metastasis before the onset of the treatment. They also underwent laparoscopic exploration again before surgery. We found that it was an important reason for achieving relatively high preoperative ORR, DCR, and R0 resection. We believe that it is essential to have the laparoscopic exploration combined with cytology as a part of the staging workup and to evaluate efficacy.

Regarding safety, all patients completed the neoadjuvant sintilimab plus XELOX therapy despite the drug dose adjustment. Leukopenia, neutropenia, and/or abnormal hepatic function were the most common hematological toxicities. Vomiting and diarrhea were the common non-hematological toxicities. The potential irAEs were hypothyroidism, pneumonia, and dermatitis. Most TRAEs were grade I to II and no treatment-related death occurred during this study. In general, preoperative addition of sintilimab to XELOX chemotherapy showed a manageable safety profile. Moreover, the proportion of operation-correlated complications was 36.7%, and the most common complications were mild pneumonia and pleural effusion without clinical symptoms. Only one patient experienced the esophageal-jejunum anastomotic leakage, which was recovered after drainage, antibiotics therapy, and nutritional support. Fortunately, no patient underwent reoperation. The surgery-related complications were also tolerable and manageable.

Meanwhile, there were several limitations in this study. First, this study was a single-arm trial without experimental group or randomization, so the selection bias could not be excluded. Second, the primary endpoint was pCR, whether the improvement of pCR will prolong patients’ OS remains to be verified by further follow-up results. Third, the sample size was relatively small and only PD-L1 expression was analyzed as a biomarker, excluding TMB, dMMR status, EBV status, and MSI status, which made it difficult for each biomarker to be correlated with the clinical efficacy.

In conclusion, these preliminary results demonstrated that sintilimab plus XELOX as a new neoadjuvant regimen showed encouraging efficacy with high pCR and MPR rate with well-tolerated safety. This combination of regimens might provide a new option for patients with LAGC. Additional large-scale clinical trials are needed to further confirm the short- and long-term efficacy of this combination of regimens.

## Data availability statement

The raw data supporting the conclusions of this article will be made available by the authors, without undue reservation.

## Ethics statement 

The study design was approved by the Ethics Committee of the Fourth Hospital of Hebei Medical University with approval number 2019125. The patients/participants provided their written informed consent to participate in this study.

## Author contributions

QZ designed the study. HG, P’aD, PY, YT, and YLiu were involved in data collection. HG, CS, and PY performed the data analysis and interpretation. HG, P’aD, and CS wrote the manuscript. SL, RB, YaL, ZZ, DW, YoL, and QZ reviewed and revised the manuscript. All authors read and approved the final manuscript.

## Funding

This work was supported by the Cultivating Outstanding Talents Project of Hebei Provincial Government Fund (No.2019012); Hebei public health committee county-level public hospitals’ suitable health technology promotion and storage project (No. 2019024) and Hebei University Science and Technology Research Project (No. ZD2019139).

## Conflict of interest

The authors declare that the research was conducted in the absence of any commercial or financial relationships that could be construed as a potential conflict of interest.

## Publisher’s note

All claims expressed in this article are solely those of the authors and do not necessarily represent those of their affiliated organizations, or those of the publisher, the editors and the reviewers. Any product that may be evaluated in this article, or claim that may be made by its manufacturer, is not guaranteed or endorsed by the publisher.
